# Wounding-Induced VOC Emissions in Five Tropical Agricultural Species

**DOI:** 10.3390/molecules26092602

**Published:** 2021-04-29

**Authors:** Miguel Portillo-Estrada, Chikodinaka N. Okereke, Yifan Jiang, Eero Talts, Eve Kaurilind, Ülo Niinemets

**Affiliations:** 1 Research Group Pleco (Plants and Ecosystems), Department of Biology, University of Antwerp, 2610 Wilrijk, Belgium; 2 Chair of Crop Science and Plant Biology, Institute of Agricultural and Environmental Sciences, Estonian University of Life Sciences, Kreutzwaldi 1, 51006 Tartu, Estonia; ChikodinakaNkechinyere.Okereke@emu.ee (C.N.O.); Eero.talts@emu.ee (E.T.); Eve.kaurilind@emu.ee (E.K.); Ylo.Niinemets@emu.ee (Ü.N.); 3 College of Horticulture, Nanjing Agricultural University, Nanjing 210095, China; jiangyifan@njau.edu.cn; 4 Estonian Academy of Sciences, Kohtu 6, 10130 Tallinn, Estonia

**Keywords:** abiotic stress, acetaldehyde, hexenal, LOX products, mass spectrometry, methanol, proton-transfer reaction, tropical crop species

## Abstract

Leaf mechanical wounding triggers a rapid release—within minutes—of a blend of volatile organic compounds. A wounding-induced VOC blend is mainly composed of oxygenated ubiquitous stress volatiles such as methanol and volatile products of lipoxygenase (LOX) pathway (mainly C5 and C6 alcohols and aldehydes and their derivatives), but also includes multiple minor VOCs that collectively act as infochemicals, inducing defences in non-damaged plant leaves and neighbouring plants and attracting herbivore enemies. At present, the interspecific variability of the rate of induction and magnitude of wounding-induced emissions and the extent to which plant structural traits and physiological activity alter these emissions are poorly known. Particularly scarce is information on the induced emissions in tropical agricultural plant species, despite their economic importance and large area of cultivation at regional and global scales. We chose five tropical crops with varying photosynthetic activity and leaf structural characteristics—*Abelmoschus esculentus*, *Amaranthus cruentus*, *Amaranthus hybridus*, *Solanum aethiopicum,* and *Telfairia occidentalis*—to characterize the kinetics and magnitude of wounding-induced emissions, hypothesizing that the induced emission response is greater and faster in physiologically more active species with greater photosynthetic activity than in less active species. Rapid highly repeatable leaf wounds (12 mm cuts) were generated by a within-leaf-chamber cutting knife. Wounding-induced VOC emissions were measured continuously with a proton-transfer reaction time-of-flight mass spectrometer and gas-chromatography mass spectrometry was used to separate isomers. Twenty-three ion VOCs and twelve terpenoid molecule structures were identified, whereas ubiquitous stress volatiles methanol (on average 40% of total emissions), hexenal (24%), and acetaldehyde (11%) were the main compounds across the species. Emissions of low-weight oxygenated compounds (LOC, 70% of total) and LOX products (29%) were positively correlated across species, but minor VOC components, monoterpenoids and benzenoids, were negatively correlated with LOC and LOX, indicating a reverse relationship between signal specificity and strength. There was a large interspecific variability in the rate of induction and emission magnitude, but the hypothesis of a stronger emission response in physiologically more active species was only partly supported. In addition, the overall emission levels were somewhat lower with different emission blend compared to the data reported for wild species, as well as different shares for the VOCs in the blend. The study demonstrates that wounding-dependent emissions from tropical agricultural crops can significantly contribute to atmospheric volatiles, and these emissions cannot be predicted based on current evidence of wild plant model systems.

## 1. Introduction

Leaf mechanical damage by piercing or chewing herbivores or strong wind or falling plant debris depicts one of the many stressors to which plants are exposed throughout their life span [1,2,3]. Wounding of the leaf under precise laboratory conditions provides an experimental setup to quantitatively study the sequence of physiological responses elicited in response to mechanical damage, e.g., damage by herbivory [4,5]. Leaf tissue wounding triggers a rapid release of a blend of volatile organic compounds (VOCs) mainly composed of short- to medium-length alcohols and aldehydes [4,5]. It contains volatile products of lipoxygenase (LOX) pathway (also called green leaf volatiles), which are the C5 and C6 alcohols and aldehydes derived from constitutive activity of different lipoxygenases, that break down polyunsaturated fatty acids released from damaged membranes [6,7]. A major methanol emission burst from damaged cell walls is also characteristic to leaf wounding [4,5,8,9] and reflects the activation of pectin methylesterases [10,11]. In addition, other low-mass oxygenated compounds (LOCs) such as formaldehyde and acetaldehyde, ketones, esters, and volatile isoprenoids [1,4,9,12,13,14,15,16,17] are released in a sequence, characterizing the activation of different pathways in different leaf compartments. As a general consequence of an open wound, temporarily, there is an uncontrolled loss of water from free water surface generated by wounding and enhanced emission of compounds that are dissolved in cell wall water and in cytosol [9]. In addition, major emission bursts can result from the damage of VOC-storing specialized cell compartments, or specialized structures in the leaf interior (e.g., resin ducts and oil glands) or on the leaf surface (glandular trichomes) [9,18].

The role of the alcohols and aldehydes emitted upon wounding is the immediate protection against herbivory by repelling the feeders, e.g., by eye and oral irritation [15,17]. The release of volatile LOX products and other wounding-associated volatiles to the surrounding air also play a signalling role, warding off or attracting herbivores or attracting herbivore natural enemies during a leaf feeding event [19]. Moreover, the synthesis of some volatiles such as (*E*)-2-hexenal and (*Z*)-3-hexenol contributes to protection against further microbial pathogen attacks through the open wound [20,21]. Synthesis of volatile plant hormones, typically jasmonic acid and its volatile methylated form, methyl jasmonate, is also induced [22,23]. Jasmonates act as emergency messengers for conspecifics and trigger the biosynthesis of benzenoids and terpenoids in leaves of the attacked plants and other neighbouring individuals [24], thereby reducing leaf palatability. From an atmospheric perspective, VOCs emitted after major leaf wounding events are released in significant amounts to the atmosphere, thereby contributing to aerosol and ozone formation, directly influencing the air quality at local to regional scales [25,26,27,28].

There is a high diversity of plant species growing within a given ecosystem and across different ecosystem types in different climatic conditions. The species differ in the activity of primary metabolism, but also have a large variation in the suites of secondary defence metabolism with important implications for the herbivore feeding behaviour. Research on the VOC emission composition, magnitude, and dynamics upon wounding has a potential to provide insight into the rate and strength of the herbivory signal in different species. Moreover, it might further be informative for resolving co-evolution strategies among plants, their feeders, and enemies of herbivores. Until present, the research on the rapid VOC emission dynamics upon leaf wounding has been primarily conducted with a few temperate climate model plants: e.g., poplars [8,12,29,30], *Arabidopsis* [31,32,33], *Dactylis glomerata* [4], and *Trifolium repens* [12]. Wound responses of agricultural plants that are grown over large areas worldwide are currently understudied, with the exception of some crops such as *Phaseolus vulgaris* [9]. This is a significant limitation, as agricultural species are potentially very vulnerable to herbivore attacks due to their high nutritive value, low secondary metabolite contents, and cultivation in monocultures [34,35]. There is particularly limited information of VOC emissions in tropical agricultural plants and about their wounding responses, despite their high contribution to worldwide vegetation cover. Given the high physiological activity of agricultural plants, including high photosynthetic and growth rates, the rate and degree of induction of defences under different stresses such as wounding stress are expected to be particularly large in agricultural plants [36]. In contrast, long-living plants in natural communities invest more in structural and chemical constitutive defences and are expected to have greater stress tolerance [36]. Nevertheless, there is a wide range of variation in photosynthetic and structural traits in crops [37,38,39]. It is expected that there is an analogous variation in induced stress responses, but to our knowledge, interspecific variation in crop induced volatile responses to mechanical stress has not yet been studied.

Given the rapid initial wounding response, fast instruments such as proton transfer reaction time-of-flight mass spectrometer (PTR-TOF-MS) are required for continuously monitoring both the emissions of wound-induced VOCs and their temporal dynamics. These recently developed instruments allow the real-time measurement of a large spectrum of VOCs (typically from 1 to 512 a.m.u.) with a time resolution of 1 to up to 10 Hz and a limit of detection under 1 ppb. The instrument is suitable for determining plant responses to rapidly occurring stresses such as wounding and measuring the overall magnitude and kinetics of the volatile emissions [4,5,12].

In this paper, we studied the wound-induced emissions in five tropical dicot crop species with contrasting photosynthetic activity. The species studied—okra (*Abelmoschus esculentus* (L.) Moench), red amaranth (*Amaranthus cruentus* L.), green amaranth (*Amaranthus hybridus* L.), African eggplant (*Solanum aethiopicum* L.), and fluted pumpkin (*Telfairia occidentalis* Hook. f.)—are currently of regional importance, but they are considered as promising future crops to diversify the food basket. *Abelmoschus esculentus*, *A. cruentus,* and *A. hybridus* are annual herbs, *S. aethiopicum* is a woody perennial but grown typically as annual herb, and *T. occidentalis* is a perennial vine. The two amaranths have C_4_ photosynthetic metabolism, and the three other species have C_3_ metabolism. Thus, we expected that there is a significant variation in foliage photosynthetic activity and structural characteristics among species. We used a PTR-TOF-MS device to assess the rate of elicitation and the magnitude of wounding-induced stress response from leaf damage until cessation of the immediate stress response. We hypothesized that both the rate of elicitation and magnitude of elicitation of the key wounding-related volatiles, methanol, and LOX pathway compounds, are greater in species with greater photosynthetic activity. We also compared the induced responses with the non-crop model species studied previously and hypothesized that the rate of induction and magnitude of elicitation is greater in the crops than in the wild species. The results provide evidence of major interspecific variability in the magnitude and kinetics of wounding-dependent emissions, indicating species-specific regulation of different stress pathways and overall demonstrating that wounding stress in crops can be a significant oxygenated volatile source.

## 2. Results

### 2.1. The Diversity in Plant Photosynthetic Capacity and Anatomical and Chemical Traits

The five studied species presented different photosynthetic capacities and stomatal conductances, and both parameters were not correlated across the species (*r*^2^ = 0.08, *p* = 0.15). *Amaranthus* species (*A. cruentus* and *A. hybridus*) with C_4_ metabolism had similar stomatal conductance, but higher photosynthetic rate than the three C_3_ species (Table 1). Mean leaf stomatal conductance and photosynthetic activity fully recovered in 30 min after wounding in all species (Student’s *t*-test at 0.05 significance level) (Table 1).

The studied species varied in both leaf structural and chemical characteristics. Leaf size varied 13-fold, with *Abelmoschus esculentus* having the largest and *Amaranthus cruentus* the smallest leaves. Leaf dry mass per area (LMA) varied two-fold, with *Telfairia occidentalis* having the greatest LMA and *Solanum aethiopicum* the lowest LMA (Table 1). Leaf N and *P* contents per area were the greatest in *Amaranthus hybridus* and *Telfairia occidentalis* (Table 1), but foliage photosynthetic N (*A*_max_/N) and photosynthetic *P* (*A*_max_/N) were the greatest in the two C_4_ species. Foliage Ca and K contents varied less among species, except *Telfairia occidentalis* had a lower K content than other species (Table 1).

The species’ structural characteristics and photosynthetic activity were generally weakly associated with wounding-elicited emissions. Nevertheless, the integrated emissions and peak maxima of the LOX product hexenal were the greatest in the two *Amaranthus* species with the highest photosynthetic rate (Table 1 and Table 2). In contrast, methanol emissions were the greatest in *Abelmoschus esculentus,* which had the largest leaves but the lowest photosynthetic rate (Table 1 and Table 2). In fact, no significant correlations were found between pre-wounding photosynthetic capacity and the integrated wound-induced emissions of total VOCs (*r*^2^ = 0.15; *p =* 0.16) nor VOC classes (e.g., LOCs: *r*^2^ = 0.22, *p =* 0.076; LOXs: *r*^2^ = 0.009, *p =* 0.74). Additionally, the baseline total VOC emissions were not correlated to photosynthetic capacity (*r*^2^ = 0.008; *p =* 0.76).

### 2.2. Wounding-Induced VOC Emissions, Dynamics, and Correlations

Total emissions upon wounding varied three-fold among the plant species, with *Amaranthus cruentus* having the lowest wounding-induced VOC emissions, and *Abelmoschus esculentus* and *Telfairia occidentalis* having the highest wounding-induced VOC emitters per mm of wound length (Table 2). The differences among species in LOCs emissions were five-fold large, and 3.7-fold in the elicitation of LOX products (Table 3).

Twenty-three VOCs were identified in the emissions from the leaves of the five studied crop species (Table 2). For most compounds, the emissions prior to leaf wounding were generally at a low level, but strongly increased after mechanical wounding. The main compounds emitted after wounding (average ± SE across species) were methanol (18 ± 6 pmol mm^−1^, 40% of total emissions), hexenal (9.3 ± 2.4 pmol mm^−1^, 24% of total), and acetaldehyde (3.7 ± 0.5 pmol mm^−1^, 11% of total), followed by formic acid (6.1 ± 1.3 pmol mm^−1^) and acetone (4.73 ± 0.15 pmol mm^−1^) (Table 2 and Table 3). The composition of the VOC blend by compound classes was: 28.5 ± 6.6 pmol mm^−1^ (70% of total) of LOCs, 10.7 ± 2.6 pmol mm^−1^ (29%) of LOXs, 0.153 ± 0.005 pmol mm^−1^ (0.5%) of benzenoids and jasmonates, 0.102 ± 0.009 pmol mm^−1^ (0.31%) of GDPP, and 0.341 ± 0.045 pmol mm^−1^ (0.97%) of isoprenoids (11 monoterpenes, 6 oxygenated monoterpenes, and 4 sesquiterpenes, Table 2 legend and Table S1, Supplementary Material) (Table 3). 

**Figure 1:**
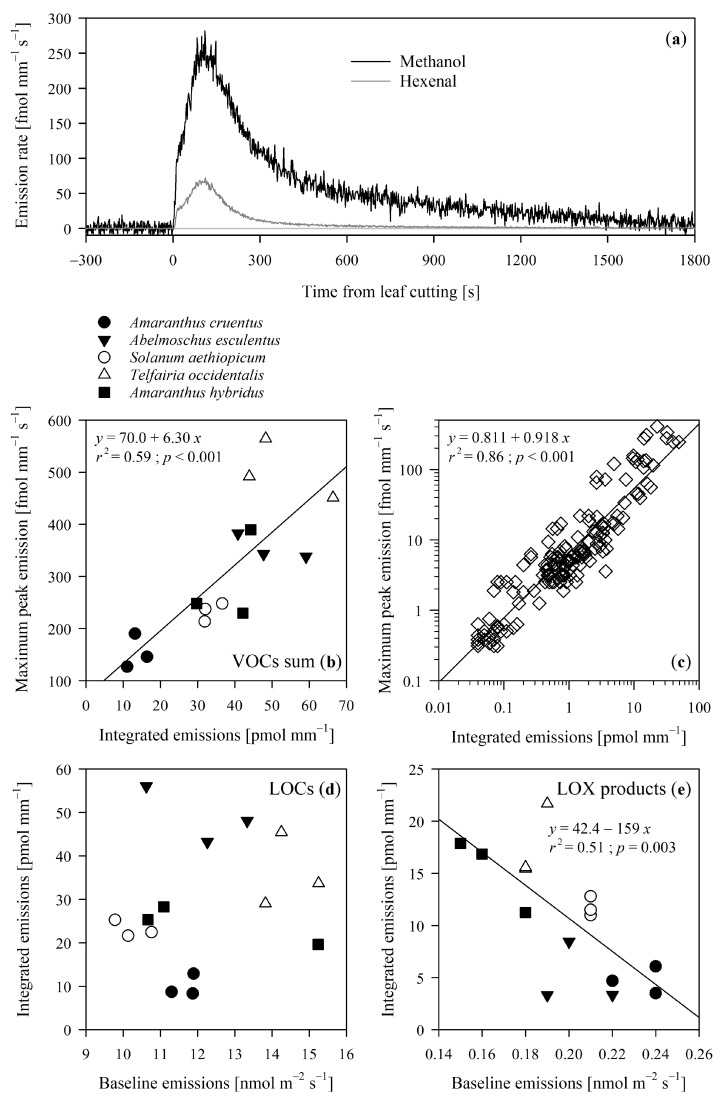
In (**a**), a representative time-course of wound-induced emissions of methanol (CH_4_O) and hexenal (C_6_H_10_O) from an *Abelmoschus esculentus* leaf. The leaf was wounded at time 0 s with a 12 mm razor cut to the lamina. In the lower panels, the correlation between peak emission and (**b**) the sum of VOCs emitted and (**c**) all the VOCs individually across five tropical agricultural species. In (**d**,**e**) the correlation between baseline emissions of LOCs and LOX products and the integrated emissions after leaf wounding. The measurements were conducted with a proton-transfer-reaction time-of-flight mass spectrometer (PTR-TOF-MS). The values shown are corrected by the leaf constitutive emissions (Table 1) by averaging the pre-wounding emission data between −300 and 0 s and subtracting it from the emission values.

VOC emissions, particularly LOCs and LOX products emissions, rapidly increased after leaf wounding until maximum values were reached, typically 1 to 2 min after wounding (see Figure 1a for a representative example of methanol and hexenal emission dynamics). Thereafter, the emissions gradually decreased to pre-wounding levels. The peak shape of the summed VOCs emitted after wounding were consistent across species (Figure 1b), as well as for the individual VOCs that peaked after wounding (Figure 1c). The VOC emission rate at the maximum varied among the VOCs (average ± SE across species), from 0.41 ± 0.05 fmol mm^−1^ s^−1^ for hexanol to 140 ± 70 fmol mm^−1^ s^−1^ for methanol (Table 2).

**Figure 2:**
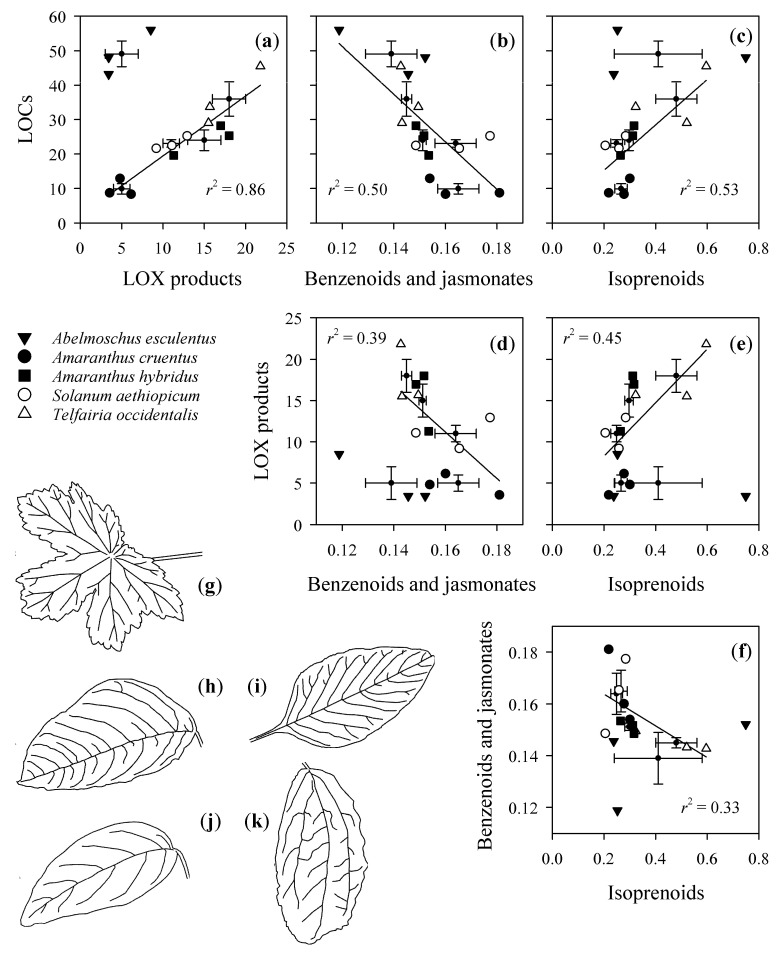
Correlations among (**a**–**f**) different classes of volatile organic compounds (VOCs) emitted [pmol mm^−1^] upon leaf wounding from leaves of five tropical crop species. Panels (**g**–**k**) show the outlines of leaves of studied species: (**g**) *Abelmoschus esculentus*, (**h**) *Amaranthus cruentus,* (**i**) *Amaranthus hybridus*, (**j**) *Telfairia occidentalis*, and (**k**) *Solanum aethiopicum*. Different symbols were used for different species. *Abelmoschus esculentus* data were excluded in correlation with LOX products and isoprenoids. Filled points with standard error bars are averages for each species. Linear correlations shown are all significant at *p =* 0.05. LOX products stands for volatile products of lipoxygenase pathway (also called green-leaf volatiles, typically C_5_-C_6_ aldehydes and alcohols, Table 1), and LOCs for low-weight oxygenated compounds (C_1_-C_3_ volatiles synthesized through diverse pathways, Table 1). Isoprenoids identification by GC-MS can be found in Table S1, Supplementary Materials.

The constitutive total VOC emissions were not correlated with the level of elicitation of VOCs after wounding (*r*^2^ = 0.22; *p =* 0.86). Similarly, no significant correlations were found when looking into VOC groups: e.g., LOCs inexistent correlation (Figure 1d) and the negative relationship exhibited between pre- and post-wound emissions of LOX products (Figure 2e).

The time to peak maximum varied among the compounds (Table 2). For example, methanol and hexenal peak maxima were found 89 ± 20 s and 43 ± 19 s after wounding (means are significantly different at *p =* 0.0066 after a paired *t*-test). In addition, significant interspecific variation in the rate of emission elicitation was found, including early emissions of methanol (16.2 ± 2.1 s) in *Telfairia occidentalis*, and late emissions of hexenal in *Abelmoschus esculentus* (98 ± 11 s) and *Amaranthus hybridus* (78 ± 10 s) compared with the emissions of these compounds in the other species (Holm–Sidak pairwise comparison among the species, *p* < 0.05). After wounding, the emissions of some volatiles in several species, e.g., monoterpenes in *Amaranthus cruentus* and *Solanum aethiopicum*, presented an enhanced emission without an identifiable peak (Table 2). Compared with LOCs and LOX products, these compounds were emitted with a lower rate (Table 2).

Despite the interspecific variation in the emission amounts and dynamics of VOC emissions, there were positive correlations (*p* < 0.05) between the integrated emissions of LOCs and isoprenoids and LOX products and isoprenoids for all species pooled—except for *Abelmoschus esculentus* (Figure 2). On the other hand, LOCs, LOX products, and isoprenoid emissions were negatively correlated with benzenoid and jasmonate emissions (Figure 2). In these relationships, *A. esculentus* was again an outlier, except for LOCs and benzenoid and jasmonate emissions (Figure 2).

## 3. Discussion

### 3.1. General Patterns in Volatile Emission

Controlled leaf mechanical damage under laboratory conditions can simulate the effects of the physical stress the plants undergo, particularly, during wounding by herbivory [4,5]. Real-time monitoring of different volatile emissions elicited by wounding can provide further insight into activation of different pathways in cell walls, membranes, and cytosol, and can indicate the degree of degradation of cell structures and oxidation of other molecules and components [4,5] as well as changes in the activity of constitutively active metabolic pathways [9]. Furthermore, precisely controlled wounding allows quantitative and qualitative comparisons of rate of different pathway elicitation and magnitude of elicitation among different species [17,40].

Typically, wounding results in defined emission peaks of key volatiles emitted, but not for all [9]. Peaked emissions indicate de novo activation of stress metabolic pathways or release of stored compounds or their non-enzymatic oxidation products after sudden exposure of cellular contents to free atmosphere, or a combination of the two processes. In the case of a gradual change of emissions, either an increase or decrease in compounds released constitutively, the wounding effect is typically indirect, through the alteration of substrate availability of compounds synthesis [9]. In the five tropical species studied, we observed both peaked and non-peaked volatile emissions after wounding, although the bulk of volatiles were released in a burst-like manner (Table 2). In fact, the induction of massive emissions of volatiles upon mechanical wounding was almost immediate: peaking in 10–15 s at 85–136 fmol mm^−1^ s^−1^ for hexenal in *A. cruentus*, *S. aethiopicum,* and *T. occidentalis,* or in 16 s at 331 fmol mm^−1^ s^−1^ for methanol in *T. occidentalis*. The emissions returned to the pre-stress level in less than 15 min for most compounds, in agreement with previous studies [5,9].

Methanol was generally the compound that peaked among the first (Table 2). The emission of methanol to wounding and to several other abiotic and biotic stresses is a ubiquitous stress response throughout all plants due to cell wall damage and associated modification in pectin demethylation [10,41,42,43]. The elicitation of methanol emissions was typically followed by LOX pathway volatiles (Table 2). The C_5_ and C_6_ compounds are emitted by wounded leaves due to the damage of cell membranes and the exposure to the atmospheric oxidant conditions [12,14]. The unsaturated fatty acids released from damaged membranes are broken down by LOX enzymes, resulting in volatile C_5_ and C_6_ compounds in the hydroperoxide lyase (HPL) branch of the oxylipin pathway [19,26,44]. These can be further acetylated to compounds such as hexyl acetate and (*Z*)-3-hexenyl acetate [19,26,44]. In our study, the first LOX products released were pentenal + 3-penten-2-one [14] and hexenal, followed by (*Z*)-3-hexen-1-ol + hexanal and (*E*)-1-hexanol, and ultimately by acetylated LOX pathway derivatives (Table 2) [12], quantitatively showing the activation of the sequence of biochemical events following mechanical damage.

Acetaldehyde was another key volatile released after leaf damage (Table 2), as observed in several other studies [5,12,25,26]. Acetaldehyde is mainly formed by enzymatic oxidation of ethanol, especially under anoxic conditions in the root zone [10,41,45], although some acetaldehyde can be directly formed from pyruvate [46,47], and there can be a certain level of C_2_ metabolites in leaves associated with fatty acid turnover. In fact, acetaldehyde was one of the compounds peaking the latest in most species (Table 2), suggesting that these emissions might be indicative of the final steps of fatty acid catabolism. On the other hand, in *A. esculentus*, there was no clear emission peak (Table 2), suggesting that wounding did not directly elicit pathways responsible for acetaldehyde release.

Apart from ubiquitous stress volatiles, we also observed the release of volatile isoprenoids and benzenoids that collectively made up a minor part of total emissions (0.49 ± 0.15% of benzenoids and 0.97 ± 0.20% of isoprenoids in all species, Table 3). Isoprene and other isoprenoids (e.g., monoterpenes, sesquiterpenes, etc.) are specialized VOCs that can be emitted in large amounts in strong constitutive emitters under non-stressed conditions [40,48,49,50]. Terpenoid emissions can be further induced under different biotic and environmental stresses, but the induction of these emissions is time-consuming, taking multiple hours to days [40,48,49,50]. The situation is analogous with volatile benzenoids (e.g., [51]). Thus, peaked emissions of volatile isoprenoids and benzenoids should indicate release from storage and non-peaked emissions indirect modifications in pathway activity. Indeed, several plant groups store different volatiles within their leaves in specialized structures (e.g., resin ducts in conifers) or on the leaf surface in glandular trichomes and upon leaf wounding. The breakage of the storage structures can expose volatiles to the atmosphere, resulting in emission bursts. We did not examine internal leaf anatomy and leaf surface characteristics in the current study, but there is evidence that all species studied have glandular trichomes on leaf surface: *A. esculentum* [52], *A. cruentus* [53], *A. hybridus* [54], *S. aethiopicum* [55], and *T. occidentalis* [56]. Different species have different density and types of glandular trichomes that store different volatile constituents [18,57], and this can be responsible for species differences in the emission dynamics of isoprenoids and benzenoids in our study. We observed that monoterpenes were released in a burst-like fashion in *A. esculentum*, *A. hybridus,* and *T. occidentalis*, whereas methyl benzoate was released in a burst-like fashion in *A. esculentum*, *A. cruentus,* and *S. aethiopicum* (Table 2).

### 3.2. Species Diversity in Emission Responses

We observed a large interspecific variation in the rate of elicitation and emission magnitude across species (Table 2)—despite a similar constitutive VOC emission level. In particular, the emission induction rate for *Amaranthus cruentus* was three-fold smaller as compared to the other amaranth, caused by comparably low methanol and hexenal emissions. Our hypothesis of the faster elicitation and greater magnitude of stress volatile induction upon wounding in physiologically more active leaves was partly supported. Indeed, hexenal emissions were elicited to a large degree in *A. hybridus*, and the fastest elicitation of hexenal emission occurred in *A. cruentus*. Both species showed the highest photosynthetic rates and highest N and P use efficiencies (*A*_max_/N and *A*_max_/P) (Table 1 and Table 2). On the other hand, methanol emissions were elicited to the greatest degree in *A. esculentus* and *T. occidentalis*, which had lower photosynthesis rates than the amaranths (Table 1 and Table 2). It is plausible that the amount of cell walls per leaf area or their thickness was greater in species with lower photosynthetic activity [58,59], but future studies are needed to test this suggestion. Clearly, our data indicate that there is no simple correlation between leaf physiological activity (e.g., *A*_max_) and wounding-induced emission response for these tropical crops. Moreover, in the same vein, constitutive volatile emissions did not correlate with the power of elicitation of VOCs upon wounding.

Despite the large interspecific variability, we observed strong positive correlations among LOCs and LOX products in the studied plant species (Figure 2), indicating coordination of the levels of elicitation for the ubiquitous volatiles, with the exception of *A. esculentus.* We observed an average ± SE ratio of LOX products/LOCs of 0.511 ± 0.039 in the emissions for the four species, whereas in *A. esculentus* this ratio was 0.104. It is plausible that the variation in this ratio reflects the overall amount of cell walls and membranes per leaf surface area and the activity of enzymes responsible for LOX products and LOCs synthesis in membranes, cell walls, and cytosol. Previous studies demonstrate that this ratio can widely vary across species: 1.23 in *Populus tremula* [5], 0.34 in *Trifolium repens*, 1.70 in *Ranunculus acris*, and 2.64 in *Dactylis glomerata* [25]. Further research is needed to assess the variation range of this ratio across more plant families.

Interestingly, we observed negative correlations among ubiquitous LOCs and LOX products and terpenoids and phenolics (Figure 2), suggesting that the magnitude of emission and specificity of the emission signal are inversely related. Given that the specialized volatiles were likely mostly coming from breakage of glandular trichomes on the leaf surface, such negative correlations might imply that the species with a greater constitutive defence capacity—i.e., greater density of glandular trichomes—have a reduced induced defence response. Previously, such a negative correlation between glandular trichome density and stress-induced volatile release was observed in ozone-stressed plants [57].

Regarding the levels of emissions in quantitative terms [pmol mm^−1^], the agricultural crop species studied here emitted relatively low levels of VOCs (e.g., 3.7 to 15.9 pmol mm^−1^ of hexenal) when wounded as compared to the 283 pmol mm^−1^ of hexenal measured in *Dactylis glomerata* [4] and 570 pmol mm^−1^ in *Populus tremula* [5]. Similarly, the emissions of methanol (2.5–40 pmol mm^−1^) and acetaldehyde (2.7–5.7 pmol mm^−1^) after wounding in the studied agricultural species were smaller than those in *Populus tremula* (76 pmol mm^-1^ for methanol and 130 pmol mm^−1^ for acetaldehyde) [5]. It could be hypothesized that the agricultural plants have been selected to have high palatability as a food source (among other characteristics including high nutritional value, low fibre content, reduction of secondary chemicals such as bitter- and astringent-tasting terpenes and phenolics), and this is related to the overall downregulation of secondary metabolic defences. Nevertheless, more research into the evolutionary determinants of VOC emissions upon wounding is needed to confirm this hypothesis.

The intraspecific variation of VOC emissions when wounding different plants was relatively low in our study (Table 2 and Table 3). In a previous study, *Populus tremula* numerous samples were taken from different leaves, and the intraspecific variability was generally low, provided that fully developed mature leaves were sampled and major veins were avoided when cutting the leaves [5,30]. Given the low intraspecific variability, we conclude that a screening study across multiple plant species can be conducted with a limited number of independent replicates. This enables large-scale investigations to find global patterns of wounding-induced VOC responses across biomes. However, such screening exercises should also consider other plant organs, because they can have different VOC compositions and emissions levels [30].

## 4. Materials and Methods

### 4.1. Plant Material and Growth Conditions

Seeds of *Abelmoschus esculentus* (L.) Moench, *Amaranthus cruentus* L.*, Amaranthus hybridus* L.*, Solanum aethiopicum* L., and *Telfairia occidentalis* Hook. f. were obtained from a private farm in Nigeria (9.1538 N, 7.3220 E, 445 m.a.s.l.) and sown in 3-litre plastic pots containing a 1:1:1 mixture of commercial potting soil with added balanced fertilizers (N:P:K = 10:8:16; Biolan Oy, Eura, Finland), quartz sand (AS Silikaat, Tallinn, Estonia), and vermiculite (Schetelig Group, Vantaa, Finland). The pH of the soil water was 6.5. Three seedlings per species were grown in an environment-controlled plant growth room. The light period was 12 h and the light intensity at plant level was 400–500 μmol m^−2^ s^−1^ (HPI-T Plus 400 W, Philips, Brussels, Belgium), day and night temperatures were 28/25 °C, and relative air humidity was 60–70%. The plants grew for 3 months before the start of the measurements. At the start of the experiments, the plants had a similar biomass, mature stem thickness, and number of mature leaves among replicate plants.

### 4.2. Leaf Photosynthetic Capacity and Stomatal Conductance

This experiment was conducted with fully developed mature attached leaves selected from the upper plant canopy. A GFS-3000 gas-exchange system (Walz GmbH, Effeltrich, Germany) with the standard cuvette enclosing 8 cm^2^ leaf area was used for photosynthetic measurements. The system was operated under the following conditions: leaf temperature of 25 °C, air relative humidity of 60%, chamber CO_2_ concentration of 400 μmol mol^−1^, incident light intensity of 500 μmol m^−2^ s^−1^ (10% blue, 90% red light), and cuvette flow rate of 750 μmol s^−1^. Outdoor air was purified by a charcoal-filled filter to minimize its background VOC concentration.

After leaf enclosure, the photosynthetic capacity (*A*_max_) and stomatal conductance (*g*_s_) were monitored during a period of not less than 45 min to ensure leaf adaptation to the chamber conditions. After full adaptation, *A*_max_ and *g*_s_ were recorded, the leaf was cut as described in the next section, and *A*_max_ and *g*_s_ were recorded again in 30 min after wounding. Right after the cutting, the water vapour signal is strongly driven by water evaporation from the cut surface, but this initial rise is short-lived and in 30 min after leaf cutting, the values of *g*_s_ accurately reflect stomatal conductance [9]. Foliage gas-exchange rates were calculated according to von Caemmerer and Farquhar [60].

### 4.3. Leaf Wounding and Volatile Organic Compound Emissions

The measurements of leaf volatile organic compound (VOC) emissions were conducted continuously with a proton-transfer-reaction time-of-flight mass spectrometer (PTR-TOF-MS) model 8000 (Ionicon Analytic GmbH, Innsbruck, Austria) following the setup and protocol of Rasulov et al. [9]. A part of the Walz GFS-3000 leaf cuvette exhaust air was diverted to PTR-TOF-MS during leaf measurements. The cuvette was specifically modified to sample the air as close as possible to the chamber, thereby avoiding any delays in VOC detection [9]. In addition, the background air VOC concentrations were measured before and after leaf enclosure. PTR-TOF-MS was operated as in Rasulov et al. [9]. In short, the drift tube field density ratio (E/N) was ≈130 Td, and the protonated ions exiting the tube were pulsed every 30 μs to the time-of-flight region, which resulted in spectra ranging from 1–278 *m/z*. A total of 60,000 spectra were averaged every 1.8 s. The PTR-MS-TOF was calibrated with a standard gas mixture containing ppm level concentrations of representatives of key volatile groups (Ionimed GmbH, Innsbruck, Austria). The data of VOCs were recorded and processed using PTR-MS Viewer v3.2 (Ionicon, Innsbruck, Austria).

Volatile organic compound (VOC) emissions together with leaf gas exchange rates were measured simultaneously through the leaf adaptation period. The emission rate for a particular VOC (Φ_VOC_; pmol m^−2^ s^−1^) using the leaf cuvette was calculated following Equation (1) (modified from Niinemets et al. [61]):

(1) $$\Phi_{\mathrm{VOC}}=\frac{\left(C_{\mathrm{VOC},\mathrm{leaf}}-C_{\mathrm{VOC},\mathrm{cuvette}}\right)\times Q_{\mathrm{cuvette}}\;}{A_{\mathrm{cuvette}}},$$

where *C*_VOC,leaf_ is the average concentration of a VOC (pmol mol^−1^) exiting the leaf cuvette, *C*_VOC,cuvette_ is the background average concentration of a VOC in the empty cuvette, *Q*_cuvette_ is the molar flow rate of the cuvette (mol s^−1^), and *A*_cuvette_ is the area of the leaf enclosed in the cuvette (m^2^). The constitutive emissions were estimated by averaging Φ_VOC_ for 5 min after the adaptation period (>150 data points, Figure 1a).

The leaf cuvette was modified by inserting a blade attached to a fine metal rod exiting the chamber (technical drawing in [9]). A sharp and quick cut of 12 mm on the leaf lamina was obtained by rotating the blade without opening the cuvette. This setup thereby avoided any contamination by ambient VOC and allowed continuous VOC measurements. The leaf cuts were made in intercostal lamina areas to avoid cutting through major veins that can result in disproportionately higher emission rates than the cuts between major veins [30]. The burst of VOC emissions after the leaf wounding was recorded for 30 min (1000 data points per compound). After this period, VOC emission levels of all compounds had typically stabilized to pre-wounding levels. The wounding stress was characterized by the integrated amount of given VOC elicited during the 30 min period expressed per wound length (pmol mm^−^^1^), as shown in Equation (2):

(2) $$\mathrm{Integrated}\;\mathrm{wound}\;\mathrm{emissions}=\frac{\sum_{time=0}^{30\;\min}\left[\left(C_{\mathrm{VOC},\mathrm{wound}}-C_{\mathrm{VOC},\mathrm{constitutive}}\right)\times Q_{\mathrm{cuvette}}\right]}{L},$$

where the concentration of a given VOC exiting the leaf cuvette after wounding, *C*_VOC,wound_, is corrected by the pre-wounding average concentration, *C*_VOC,constitutive_, referred to the cuvette’s molar flow, *Q*_cuvette_, and normalized by cut length (*L*; mm).

The wounding VOC emission peaks were fitted by a bi-Gaussian function following the protocol in [5] due to the longer tail after the maximum. A peak-like emission pattern was considered when the emission data successfully fitted the Gaussian equation at *p* = 0.05 level. The maximum emission rate (fmol mm^−^^1^ cut length s^−^^1^) was given by the absolute maximum of the peak fit, and the time from wounding to the peak emission (s) was also reported. All experiments were replicated at least thrice for each species with independent plants.

The emitted VOCs were grouped into five classes according to [5]:

- Lightweight Oxygenated Compounds (LOCs): formaldehyde, methanol, acetaldehyde, formic acid, ethanol, acetone, acetic acid;
- Volatile products of lipoxygenase pathway (LOX products): pentenal + 3-penten-2-one, pentanal + 2-pentanone, hexenal, (*Z*)-3-hexen-1-ol + hexanal, (*E*)-1-hexanol, hexyl acetate;
- Geranylgeranyl diphosphate (GGDP) pathway—6-methyl-5-hepten-2-one;
- Benzenoids and jasmonates—methyl benzoate, methyl salicylate, jasmonic acid, methyl jasmonate;
- Isoprenoids—non-oxygenated monoterpenes, (*E*)-4,8-dimethyl-1,3,7-nonatriene (DMNT), oxygenated monoterpenes, sesquiterpenes, and (*E*,*E*)-4,8,12-trimethyltrideca-1,3,7,11-tetraene (TMTT). The mass ions used to identify different volatiles are provided in Table 2. Identity of compounds with C_2_-C_15_ was verified by GC-MS (Table S1; Supplementary Material). Although all studied species emitted trace levels of isoprene [48], isoprene and some C_5_ LOX products cannot be separated during wounding by this setup.

### 4.4. Compound Verification by Gas Chromatography Mass Spectrometry (GC-MS)

GC-MS analysis was used to verify the identity of compounds emitted constitutively and during wounding. Volatiles were collected from the cuvette exhaust air through a Teflon T-piece and PTFE tubing with a 210–1003 MTX (SKC Inc., Houston, TX, USA) air sample pump with a rate of 0.2 L min^−1^ for 20 min, resulting in sampling of 4 L of air. Volatiles were collected onto multi-bed stainless steel cartridges filled with three different carbon-based adsorbents—Carbotrap C 20/40 mesh, Carbopack B 40/60 mesh, and Carbotrap X20/40 (Supelco, Bellefonte, PA, USA)—for optimal adsorption of all volatiles between C_3_ and C_17_ [62]. Blank samples were taken from the cuvette air without the leaf. The analysis of cartridges was carried out with a combined Shimadzu TD20 automated cartridge desorber and a Shimadzu 2010 Plus GC-MS (Shimadzu Corporation, Kyoto, Japan), as described in detail in [62,63,64]. A quantitative analysis of lightweight oxygenated volatiles, fatty acid derived compounds, volatile isoprenoids, monoterpenes, and sesquiterpenes was performed to confirm the structure of the ions measured by PTR-TOF-MS. GC-MS was calibrated using authentic standards (Sigma-Aldrich, St. Louis, MO, USA) for key LOX, mono- and sesquiterpenes, and benzenoids, as described in detail in Kännaste et al. [62]. The identification of compounds was done on the basis of authentic standards and compound retention times and mass spectra using NIST 14 spectral library using the open-access program OpenChrom ver 1.2.0 (Alder) (Lablicate GmbH, Hamburg, Germany) [36]. Three replicate measurements per plant species were conducted (Table S1).

## 5. Conclusions

The study demonstrates that wounding-dependent emissions from tropical agricultural crops are a significant source of oxygenated volatiles, can significantly contribute to atmospheric volatiles, and their emissions cannot be predicted based on current evidence of wild plant model systems.

The hypothesis of a stronger emission response in physiologically more active species (highest *A*_max_/N and *A*_max_/P) was only partly supported by the amaranths.

The wounding stress emissions were dominated by methanol (40 ± 9% of total emissions), hexenal (24.5 ± 4.4%), and acetaldehyde (11.0 ± 2.5%) across the species. Emissions of low-weight oxygenated compounds (LOC, 70% of total) and LOX products (29%) were positively correlated across species. However, minor VOC components, monoterpenoids and benzenoids, were negatively correlated with LOC and LOX, indicating a reverse relationship between signal specificity and strength.

## Figures and Tables

**Table 1:** Average ± SE (standard error) leaf photosynthetic and structural characteristics and contents of macroelements.

Characteristic	Units	*Abelmoschus esculentus*	*Amaranthus cruentus*	*Amaranthus hybridus*	*Solanum aethiopicum*	*Telfairia occidentalis*
Photosynthesis rate before cut	μmol m^−2^ s^−1^	4.6 ± 1.2 ^a^	10.7 ± 1.0 ^ab^	11.8 ± 1.6 ^b^	5.7 ± 1.4 ^ab^	6.2 ± 2.2 ^ab^
Photosynthesis rate after cut	μmol m^−2^ s^−1^	3.6 ± 1.1 ^a^	11.1 ± 1.0 ^b^	11.6 ± 1.9 ^b^	5.1 ± 1.2 ^ab^	5.5 ± 1.9 ^ab^
Stomatal conductance before cut	mmol m^−2^ s^−1^	66 ± 11 ^a^	83 ± 8 ^ab^	125 ± 6 ^bc^	175 ± 23 ^c^	89 ± 5 ^ab^
Stomatal conductance after cut	mmol m^−2^ s^−1^	65 ± 24 ^a^	85 ± 10 ^ab^	110 ± 11 ^ab^	146 ± 7 ^b^	88 ± 9 ^ab^
Dry mass	Mg	1050 ± 130 ^b^	77 ± 7 ^a^	119 ± 9 ^a^	230 ± 70 ^a^	275 ± 33 ^a^
Leaf area	cm^2^	290 ± 38 ^b^	28.2 ± 1.8 ^a^	30.8 ± 1.9 ^a^	86.1 ± 3.1 ^a^	58 ± 10 ^a^
Leaf dry mass per unit area	g m^−2^	36.30 ± 0.46	27.1 ± 0.6	38.5 ± 0.6	27 ± 8	53 ± 17
Pre-wound *A*_max_/N	μmol s^−1^ g^−1^	5.3 ± 2.3	11.0 ± 1.1	10.9 ± 1.4	4.6 ± 1.6	6.6 ± 1.6
Pre-wound *A*_max_/P	μmol s^−1^ g^−1^	23 ± 10	44.7 ± 4.4	34.1 ± 4.5	20 ± 7	26 ± 6
Nitrogen	g m^−2^	0.750 ± 0.018 ^a^	0.973 ± 0.010 ^c^	1.083 ±0.012 ^d^	0.868 ± 0.024 ^b^	1.337 ± 0.027 ^e^
Carbon	g m^−2^	15.07 ± 0.11 ^b^	10.07 ± 0.08 ^a^	14.34 ± 0.15 ^b^	10.57 ± 0.23 ^a^	21.3 ± 0.8 ^c^
Phosphorus	g m^−2^	0.173 ± 0.006 ^a^	0.239 ± 0.008 ^b^	0.348 ± 0.007 ^d^	0.219 ± 0.006 ^b^	0.310 ± 0.006 ^c^
Potassium	g m^−2^	1.855 ± 0.034 ^c^	1.647 ± 0.030 ^b^	1.681 ± 0.020 ^b^	1.549 ± 0.020 ^b^	1.148 ± 0.041 ^a^
Calcium	g m^−2^	1.120 ± 0.023 ^c^	0.656 ± 0.006 ^a^	0.721 ± 0.005 ^b^	0.952 ± 0.009 ^c^	0.725 ± 0.008 ^b^
Magnesium	g m^−2^	0.633 ± 0.018 ^e^	0.499 ± 0.014 ^d^	0.564 ± 0.016 ^c^	0.248 ± 0.007 ^a^	0.383 ± 0.007 ^b^

*A*_max_ is photosynthetic capacity at saturating light and optimal conditions. Different superscript letters next to the values (a–e) indicate significant differences among species after Tukey *HSD* (honestly significant difference) test (*p* < 0.05). No significant differences were found between pre- and post-wounding (30 min after leaf wounding) leaf photosynthesis rate and stomatal conductance (*p* > 0.05 according to paired samples *t*-test; *n* = 3 for all species).

**Table 2:** Volatile organic compound (VOC) emissions (average ± SE) of five tropical crop species in (1) at optimal conditions of light, air temperature and moisture (constitutive emissions), and (2) after a 12 mm cut was made into the leaf lamina. The VOC emission rate (3) at the wounding-induced peak maximum and (4) the time to the peak maximum are also provided when the pattern of VOC emissions after wounding included a peak. Information on individual contributions to the total emissions in percentage is in Table 3.

Compound Name	Molecular Formula	Protonated Molecular Mass	(1) Constitutive Emissions (2) Wound Emissions (3) Max. Emission Rate (4) Time from Wounding	*Abelmoschus esculentus*	*Amaranthus cruentus*	*Amaranthus hybridus*	*Solanum aethiopicum*	*Telfairia occidentalis*
Formaldehyde	CH_2_O	31.0178	pmol m^−2^ s^−1^	740 ± 80	612.3 ± 3.7	566 ± 41	542.6 ± 4.1	749 ± 7
			pmol mm^−1^	0.65 ± 0.14	0.765 ± 0.022	0.75 ± 0.08	0.753 ± 0.049	0.66 ± 0.07
			fmol mm^−1^ s^−1^	-	-	-	-	-
			s	-	-	-	-	-
Methanol	CH_4_O	33.0335	pmol m^−2^ s^−1^	1600 ± 100	1553 ± 28	1270 ± 160	1750 ± 250	2300 ± 140
			pmol mm^−1^	40.2 ± 4.8	2.52 ± 0.33	14.1 ± 2.0	11.3 ± 2.6	23.2 ± 4.7
			fmol mm^−1^ s^−1^	275 ± 30	16.0 ± 1.6	46.7 ± 4.8	47 ± 8	331 ± 49
			s	75 ± 31	111 ± 16	130 ± 17	112 ± 10	16.2 ± 2.1
Acetaldehyde	C_2_H_4_O	45.0335	pmol m^−2^ s^−1^	4020 ± 330	3990 ± 90	5650 ± 1000	2750 ± 150	5310 ± 260
			pmol mm^−1^	2.74 ± 0.20	3.0 ± 1.3	3.40 ± 0.09	3.88 ± 0.53	5.7 ± 0.6
			fmol mm^−1^ s^−1^	-	21.9 ± 2.1	15.9 ± 0.8	14.7 ± 1.8	17.5 ± 2.6
			s	-	733 ± 45	123 ± 32	639 ± 37	461 ± 35
Formic acid	CH_2_O_2_	47.0128	pmol m^−2^ s^−1^	2550 ± 70	2895 ± 30	2390 ± 60	2810 ± 80	2473 ± 19
			pmol mm^−1^	1.73 ± 0.1	1.62 ± 0.08	2.41 ± 0.40	2.34 ± 0.27	2.2 ± 0.6
			fmol mm^−1^ s^−1^	-	6.3 ± 1.0	10.4 ± 1.9	7.3 ± 0.9	10.9 ± 1.3
			s	-	119 ± 21	158 ± 18	205 ± 40	96 ± 8
Ethanol	C_2_H_6_O	47.0491	pmol m^−2^ s^−1^	622 ± 240	404 ± 31	412 ± 22	396.2 ± 4.4	457 ± 28
			pmol mm^−1^	0.728 ± 0.028	0.56 ± 0.13	0.68 ± 0.11	1.7 ± 1.0	0.80 ± 0.18
			fmol mm^−1^ s^−1^	2.813 ± 0.023	3.1 ± 0.7	3.13 ± 0.10	2.8 ± 0.05	3.44 ± 0.26
			s	78.3 ± 3.7	33 ± 7	193.5 ± 3.7	297 ± 22	100 ± 11
Acetone	C_3_H_6_O	59.0491	pmol m^−2^ s^−1^	1430 ± 430	864 ± 5	1160 ± 410	795 ± 75	2130 ± 210
			pmol mm^−1^	2.3 ± 0.8	0.75 ± 0.08	1.9 ± 0.7	1.7 ± 0.6	2.6 ± 0.7
			fmol mm^−1^ s^−1^	5.6 ± 1.0	5.0 ± 1.7	6.3 ± 0.6	5.6 ± 2.0	7.9 ± 1.3
			s	182 ± 44	15 ± 5	123 ± 13	120 ± 50	339 ± 150
Acetic acid	C_2_H_4_O_2_	61.0284	pmol m^−2^ s^−1^	1110 ± 90	1370 ± 80	881 ± 26	1800 ± 100	1010 ± 50
			pmol mm^−1^	0.77 ± 0.10	0.71 0.15	1.17 0.23	1.38 0.20	0.77 0.16
			fmol mm^−1^ s^−1^	5.3 ± 1.8	5.00 ± 0.36	4.6 ± 0.6	4.7 ± 1.3	4.6 ± 1.0
			s	83 ± 35	49 ± 30	150 ± 42	172 ± 65	56 ± 22
Pentenal + 3-penten-2-one	C_5_H_8_O	85.0648	pmol m^−2^ s^−1^	21.4 ± 1.1	28.7 ± 0.8	17.9 ± 1.5	21.9 ± 0.7	21.2 ± 0.6
			pmol mm^−1^	0.203 ± 0.034	0.43 ± 0.09	0.64 ± 0.05	0.56 ± 0.15	0.55 ± 0.09
			fmol mm^−1^ s^−1^	4.2 ± 0.9	9.8 ± 2.5	5.63 ± 0.36	12.5 ± 3.2	4.8 ± 0.9
			s	11.1 ± 1.8	9.6 ± 2.4	78 ± 12	10.8 ± 1.8	26.4 ± 3.9
Pentanal + 2-pentanone	C_5_H_10_O	87.0804	pmol m^−2^ s^−1^	89.3 ± 2.5	109.8 ± 2.4	75.4 ± 3.3	96.2 ± 1.4	85.7 ± 4.5
			pmol mm^−1^	0.252 ± 0.026	0.302 ± 0.015	0.386 ± 0.017	0.333 ± 0.022	0.390 ± 0.011
			fmol mm^−1^ s^−1^	-	-	-	-	-
			s	-	-	-	-	-
Hexenal	C_6_H_10_O	99.0804	pmol m^−2^ s^−1^	41 ± 11	36.3 ± 0.9	24.5 ± 2.5	32.6 ± 2.4	28.9 ± 1.1
			pmol mm^−1^	4.3 ± 1.6	3.7 ± 0.7	13.1 ± 1.7	9.7 ± 0.9	15.9 ± 1.9
			fmol mm^−1^ s^−1^	78 ± 35	85 ± 17	188 ± 43	136.7 ± 4.5	117 ± 8
			s	98 ± 11	9.6 ± 0.6	78 ± 10	15.0 ± 1.6	14.4 ± 2.1
(*Z*)-3-hexen-1-ol + hexanal	C_6_H_12_O	101.0961	pmol m^−2^ s^−1^	39.4 ± 2.6	48.7 ± 2.2	36.9 ± 1.6	48.4 ± 1.6	37.0 ± 1.7
			pmol mm^−1^	0.210 ± 0.44	0.2045 ± 0.0042	1.11 ± 0.31	0.27 ± 0.06	0.65 ± 0.11
			fmol mm^−1^ s^−1^	1.88 ± 0.50	-	5.0 ± 1.3	-	3.54 ± 0.21
			s	122 ± 11	-	186 ± 8	-	70 ± 19
(*E*)-1-hexanol	C_6_H_14_O	103.1117	pmol m^−2^ s^−1^	5.68 ± 0.20	6.49 ± 0.22	4.8 ± 0.7	5.58 ± 0.30	5.58 ± 0.40
			pmol mm^−1^	0.054 ± 0.006	0.0481 ± 0.0026	0.053 ± 0.005	0.043 ± 0.005	0.052 ± 0.005
			fmol mm^−1^ s^−1^	0.344 ± 0.026	0.52 ± 0.06	-	0.38 ± 0.05	-
			s	293 ± 18	304 ± 23	-	311 ± 18	-
Methyl benzoate	C_8_H_8_O_2_	137.0597	pmol m^−2^ s^−1^	5.6 ± 1.0	7.0 ± 0.8	4.1 ± 0.5	4.8 ± 0.7	4.21 ± 0.28
			pmol mm^−1^	0.059 ± 0.008	0.081 ± 0.010	0.0614 ± 0.0033	0.076 ± 0.007	0.0573 ± 0.0041
			fmol mm^−1^ s^−1^	0.38 ± 0.07	0.65 ± 0.06	-	0.50 ± 0.08	-
			s	284 ± 13	317 ± 18	-	322 ± 16	-
Non-oxygenated monoterpenes ^(a)^	C_10_H_16_	137.1325	pmol m^−2^ s^−1^	44 ± 13	33 ± 6	42 ± 8	32 ± 7	48 ± 12
			pmol mm^−1^	0.25 ± 0.17	0.092 ± 0.011	0.094 ± 0.009	0.066 ± 0.021	0.32 ± 0.08
			fmol mm^−1^ s^−1^	3.1 ± 1.0	-	2.5 ± 0.8	-	1.67 ± 0.42
			s	130 ± 90	-	387 ± 22	-	360 ± 120
6-Methyl-5-hepten-2-one	C_8_H_14_O_2_	143.1067	pmol m^−2^ s^−1^	24.2 ± 1.3	28.05 ± 0.40	19.5 ± 0.9	26.9 ± 1.6	22.1 ± 0.5
			pmol mm^−1^	0.089 ± 0.008	0.084 ± 0.014	0.124 ± 0.022	0.124 ± 0.012	0.089 ± 0.009
			fmol mm^−1^ s^−1^	-	-	-	-	-
			s	-	-	-	-	-
Hexyl acetate	C_8_H_16_O_2_	145.1223	pmol m^−2^ s^−1^	4.44 ± 0.21	4.93 ± 0.13	3.97 ± 0.12	4.69 ± 0.28	3.969 ± 0.017
			pmol mm^−1^	0.0357 ± 0.0039	0.0402 ± 0.0023	0.0334 ± 0.0005	0.0341 ± 0.0031	0.0366 ± 0.0019
			fmol mm^−1^ s^−1^	-	0.41 ± 0.08	0.594 ± 0.026	-	-
			s	-	338 ± 47	520 ± 90	-	-
DMNT	C_11_H_18_	151.1481	pmol m^−2^ s^−1^	5.41 ± 0.27	6.72 ± 0.12	5.416 ± 0.035	6.1 ± 0.7	5.42 ± 0.12
			pmol mm^−1^	0.041 ± 0.006	0.043 ± 0.006	0.040 ± 0.005	0.053 ± 0.011	0.0385 ± 0.0020
			fmol mm^−1^ s^−1^	-	-	-	-	-
			s	-	-	-	-	-
Methyl salicylate	C_8_H_8_O_3_	153.0546	pmol m^−2^ s^−1^	6.1 ± 1.0	5.17 ± 0.26	3.50 ± 0.10	4.5 ± 0.5	5.2 ± 1.1
			pmol mm^−1^	0.0272 ± 0.0041	0.0330 ± 0.0049	0.0426 ± 0.0013	0.0386 ± 0.0036	0.0351 ± 0.0011
			fmol mm^−1^ s^−1^	-	-	-	-	-
			s	-	-	-	-	-
Oxygenated monoterpenes ^(a)^	C_10_H_18_O	155.1430	pmol m^−2^ s^−1^	4.08 ± 0.08	4.73 ± 0.43	5.8 ± 2.7	4.04 ± 0.21	3.90 ± 0.43
			pmol mm^−1^	0.0521 ± 0.0022	0.049 ± 0.006	0.089 ± 0.010	0.0534 ± 0.0019	0.047 ± 0.006
			fmol mm^−1^ s^−1^	-	-	-	-	-
			s	-	-	-	-	-
Sesquiterpenes ^(a)^	C_15_H_24_	205.1951	pmol m^−2^ s^−1^	6.19 ± 0.35	9.21 ± 0.21	6.3 ± 0.6	6.96 ± 0.17	6.9 ± 1.5
			pmol mm^−1^	0.0357 ± 0.0018	0.0472 ± 0.0049	0.0341 ± 0.0029	0.0391 ± 0.0006	0.041 ± 0.011
			fmol mm^−1^ s^−1^	-	-	-	-	-
			s	-	-	-	-	-
Jasmonic acid	C_12_H_18_O_3_	211.1329	pmol m^−2^ s^−1^	1.845 ± 0.022	2.286 ± 0.026	1.84 ± 0.16	2.02 ± 0.15	1.77 ± 0.17
			pmol mm^−1^	0.0263 ± 0.0019	0.0221 ± 0.0010	0.0234 ± 0.0014	0.0243 ± 0.0035	0.0247 ± 0.0029
			fmol mm^−1^ s^−1^	-	-	-	-	-
			s	-	-	-	-	-
TMTT	C_16_H_26_	219.2107	pmol m^−2^ s^−1^	5.84 ± 0.06	7.55 ± 0.20	4.78 ± 0.28	6.13 ± 0.19	5.25 ± 0.24
			pmol mm^−1^	0.0350 ± 0.0032	0.0344 ± 0.0044	0.0396 ± 0.0019	0.038 ± 0.007	0.0373 ± 0.0017
			fmol mm^−1^ s^−1^	-	-	-	-	-
			s	-	-	-	-	-
Methyl jasmonate	C_13_H_20_O_3_	225.1485	pmol m^−2^ s^−1^	2.38 ± 0.19	2.371 ± 0.044	2.56 ± 0.13	2.52 ± 0.11	2.21 ± 0.13
			pmol mm^−1^	0.0265 ± 0.0021	0.0292 ± 0.0018	0.0239 ± 0.0018	0.0246 ± 0.0031	0.0280 ± 0.0007
			fmol mm^−1^ s^−1^	-	-	-	-	-
			s	-	-	-	-	-
Total constitutive emissions			nmol m^−2^ s^−1^	12.03 ± 0.19	12.6 ± 1.5	12.4 ± 0.8	10.53 ± 0.30	14.73 ± 0.43
Total wound emissions			pmol mm^−1^	55 ± 8	15.2 ± 2.9	40 ± 6	35 ± 6	54 ± 9

The measurements were conducted with a proton-transfer-reaction time-of-flight mass spectrometer (PTR-TOF-MS) for 5 min before (constitutive emissions) and for 30 min after wounding (integrated emissions, peak emissions). Abbreviations: DMNT-(*E*)-4,8-dimethyl-1,3,7-nonatriene; TMTT-(*E*,*E*)-4,8,12-trimethyltrideca-1,3,7,11-tetraene. ^(a)^ Thermal desorption gas-chromatography mass-spectrometry (GC-MS) analysis was used to confirm the identity of emitted compounds and analyse the composition of released volatiles classes (see Table S1, Supplementary Material). GC-MS analysis demonstrated that monoterpenes (C_10_H_16_) were composed of camphene, Δ^3^-carene, *p*-cymene, limonene, *α*-myrcene, *β*-ocimene, *α*-pinene, *β*-pinene, *α*-phellandrene, *β*-phellandrene, and *α*-terpinene; oxygenated monoterpenes (C_10_H_18_O) were composed of (*E*)-dihydrocarvone, camphor, *β*-cyclocytral, 1,8-cineole, linalool, and *α*-terpineol; and sesquiterpenes (C_15_H_24_) were composed of cubebene, *(E*)-*β*-farnesene, longifolene, and aromadendrene.

**Table 3:** Chemical composition of the overall blend of VOCs emitted after a 12 mm cut was made into the leaf lamina. Values are expressed as percentage contribution to the total emissions, and in pmol mm^−1^ in the summary per VOC classes. More emission peak information is found in Table 2.

Compound Name	Molecular Formula	*Abelmoschus esculentus*	*Amaranthus cruentus*	*Amaranthus hybridus*	*Solanum aethiopicum*	*Telfairia occidentalis*	Mean ± SE
Lightweight oxygenated compounds (LOCs)		49 ± 6 pmol mm^−1^	9.9 ± 2.1 pmol mm^−1^	24.4 ± 3.6 pmol mm^−1^	23 ± 5 pmol mm^−1^	36 ± 7 pmol mm^−1^	29 ± 7 pmol mm^−1^
Formaldehyde	CH_2_O	1.2%	5.0%	1.9%	2.2%	1.2%	2.3 ± 0.7%
Methanol	CH_4_O	74%	17%	35%	33%	43%	40 ± 9%
Acetaldehyde	C_2_H_4_O	5%	20%	9%	11%	11%	11.0 ± 2.5%
Formic acid	CH_2_O_2_	3.2%	11%	6%	7%	4.1%	6.1 ± 1.3%
Ethanol	C_2_H_6_O	1.3%	3.7%	1.7%	4.9%	1.5%	2.6 ± 0.7%
Acetone	C_3_H_6_O	4.1%	5.0%	4.8%	5.0%	4.9%	4.74 ± 0.15%
Acetic acid	C_2_H_4_O_2_	1.4%	4.7%	2.9%	4.0%	1.4%	2.9 ± 0.7%
Total		90%	66%	61%	67%	66%	70 ± 5%
Lipoxygenase pathway products (LOX products)		5.0 ± 1.7 pmol mm^−1^	4.7 ± 0.8 pmol mm^−1^	15.3 ± 2.1 pmol mm^−1^	10.9 ± 1.1 pmol mm^−1^	17.6 ± 2.1 pmol mm^−1^	10.7 ± 2.6 pmol mm^−1^
Pentenal + 3-penten-2-one	C_5_H_8_O	0.37%	2.8%	1.6%	1.6%	1.0%	1.49 ± 0.41%
Pentanal + 2-pentanone	C_5_H_10_O	0.46%	2.0%	1.0%	1.0%	0.7%	1.02 ± 0.26%
Hexenal	C_6_H_10_O	8%	25%	33%	28%	29%	24.5 ± 4.4%
(*Z*)-3-hexen-1-ol + hexanal	C_6_H_12_O	0.38%	1.3%	2.8%	0.8%	1.2%	1.29 ± 0.40%
(*E*)-1-hexanol	C_6_H_14_O	0.10%	0.32%	0.13%	0.13%	0.10%	0.154 ± 0.042%
Hexyl acetate	C_8_H_16_O_2_	0.07%	0.27%	0.08%	0.10%	0.07%	0.116 ± 0.038%
Total		9%	31%	38%	32%	32%	29 ± 5%
Benzenoids and jasmonates		0.139 ± 0.016 pmol mm^−1^	0.165 ± 0.018 pmol mm^−1^	0.151 ± 0.008 pmol mm^−1^	0.164 ± 0.017 pmol mm^−1^	0.145 ± 0.009 pmol mm^−1^	0.153 ± 0.005 pmol mm^−1^
Methyl benzoate	C_8_H_8_O_2_	0.11%	0.53%	0.15%	0.22%	0.11%	0.22 ± 0.08%
Methyl salicylate	C_8_H_8_O_3_	0.05%	0.22%	0.11%	0.11%	0.06%	0.110 ± 0.029%
Jasmonic acid	C_12_H_18_O_3_	0.048%	0.15%	0.06%	0.07%	0.045%	0.074 ± 0.019%
Methyl jasmonate	C_13_H_20_O_3_	0.048%	0.19%	0.06%	0.07%	0.05%	0.085 ± 0.027%
Total		0.25%	1.1%	0.38%	0.47%	0.27%	0.49 ± 0.15%
Geranylgeranyl diphosphate pathway		0.089 ± 0.008 pmol mm^−1^	0.084 ± 0.014 pmol mm^−1^	0.124 ± 0.022 pmol mm^−1^	0.124 ± 0.012 pmol mm^−1^	0.089 ± 0.009 pmol mm^−1^	0.102 ± 0.009 pmol mm^−1^
6-Methyl-5-hepten-2-one	C_8_H_14_O_2_	0.16%	0.55%	0.31%	0.36%	0.16%	0.31 ± 0.7%
Isoprenoids		0.41 ± 0.18 pmol mm^−1^	0.266 ± 0.032 pmol mm^−1^	0.297 ± 0.029 pmol mm^−1^	0.249 ± 0.041 pmol mm^−1^	0.48 ± 0.10 pmol mm^−1^	0.341 ± 0.045 pmol mm^−1^
Non-oxygenated monoterpenes ^(a)^	C_10_H_16_	0.46%	0.6%	0.23%	0.19%	0.6%	0.41 ± 0.09%
DMNT	C_11_H_18_	0.07%	0.28%	0.10%	0.15%	0.07%	0.136 ± 0.039%
Oxygenated monoterpenes ^(a)^	C_10_H_18_O	0.10%	0.32%	0.22%	0.15%	0.09%	0.176 ± 0.044%
Sesquiterpenes ^(a)^	C_15_H_24_	0.07%	0.31%	0.08%	0.11%	0.08%	0.130 ± 0.046%
TMTT	C_16_H_26_	0.06%	0.23%	0.10%	0.11%	0.07%	0.114 ± 0.030%
Total		0.75%	1.8%	0.7%	0.7%	0.9%	0.97 ± 0.20%

^(a)^ GC-MS analysis revealed the compound structures composing these ion masses (Table S1, Supplementary Material).

## Data Availability

The data used in this article is publicly available in a digital repository: https://doi.org/10.6084/m9.figshare.14393549.v2 (accessed on 28 April 2021).

## Supplementary Materials

The following are available online, Table S1: Average (±SE; *n* = 3) constitutive VOC emission rates [nmol m^−2^ s^−1^] in five tropical crop species measured by gas chromatography.

## Sample Availability

Samples of the compounds are not available from the authors.
