# Supplementary material for: Wounding-Induced VOC Emissions in Five Tropical Agricultural Species
Source: Molecules. 2021 Apr 29;26(9):2602. doi: 10.3390/molecules26092602 (PMC8125398; doi:10.3390/molecules26092602)
Supplement: Supplementary file 1 [file molecules-26-02602-s001.zip › molecules-1186365-supplementary.pdf]

Supplementary Material

Table S1. Average (± standard error; *n* = 3) constitutive VOC emission rates [nmol m^{-2} s^{-1}] in five tropical crop species measured by gas chromatography.

Compound class	Compound name	Molecular formula	***Abelmoschus esculentus***	***Amaranthus cruentus***	***Amaranthus hybridus***	***Solanum aethiopicum***	***Telfairia occidentalis***
**Lightweight oxygenated compounds (LOCs)**	Acetaldehyde	C_2H_4O	42 ± 22	45 ± 23	55 ± 55	23 ± 23	48 ± 39
	Acetone	C_3H_6O	155 ± 65	208 ± 91	70 ± 37	68 ± 53	105 ± 65
**Lipoxygenase pathway products (LOX products)**	1-Penten-3-one	C_5H_8O	nd	nd	3.5 ± 2.2	1.2 ± 1.2	3.8 ± 3.3
	Pentanal	$C_5H_{10}O$	10 ± 8	16 ± 5.7	20 ± 12	nd	3.0 ± 2.4
	2-Pentanone	$C_5H_{10}O$	7 ± 5	2.4 ± 2.26	7.3 ± 2.1	nd	6.1 ± 3.9
	1-Pentanol	$C_5H_{12}O$	nd	nd	2.1 ± 1.7	3.9 ± 3.4	0.090 ± 0.085
	1-Penten-3-ol	$C_5H_{12}O$	nd	nd	32 ± 16	nd	nd
	(*Z*)-3-Hexen-1-ol	$C_6H_{12}O$	nd	nd	21 ± 20	nd	nd
	(*E*)-3-Hexen-1-ol	$C_6H_{12}O$	nd	nd	2.2 ± 2.2	nd	nd
	Hexanal	$C_6H_{12}O$	28 ± 12	12 ± 12	34 ± 7.7	31 ± 15	30 ± 15
	(*E*)-1-hexanol	$C_6H_{14}O$	38 ± 14	44 ± 15	20 ± 6.5	11 ± 8.6	27 ± 14
**Fatty acid derived compounds**	Heptanal	$C_7H_{14}O$	14 ± 4.6	21 ± 2.5	36 ± 18	nd	2.4 ± 1.9
	Octanal	$C_8H_{16}O$	13 ± 3.3	41 ± 5.7	41 ± 31	nd	3.2 ± 3.2
	Nonanal	$C_9H_{18}O$	42 ± 21	93 ± 23	98 ± 77	15 ± 15	4.6 ± 2.9
	Decanal	$C_{10}H_{20}O$	92 ± 16	40 ± 26	71 ± 61	2.4 ± 2.4	2.0 ± 2.0
**Benzenoids**	Benzaldehyde	$C_8H_8O_2$	10 ± 3.5	34 ± 9.1	28 ± 4.9	19 ± 19	46 ± 21
**Geranylgeranyl diphosphate pathway**	6-Methyl-5-hepten-2-one	$C_8H_{14}O_2$	48 ± 8	20 ± 4.4	66 ± 41	45 ± 45	40 ± 37
	Geranyl acetone	$C_{13}H_{22}O$	21 ± 13	47 ± 29	24 ± 12	nd	3.2 ± 3.2

**Hemiterpene**	Isoprene	C_5H_8	52 ± 21	110 ± 19	48 ± 35	43 ± 17	69 ± 43
**Non-oxygenated monoterpenes**	Camphene	$C_{10}H_{16}$	nd	13 ± 5.3	nd	0.92 ± 0.92	4.4 ± 3.9
	Δ^3-Carene	$C_{10}H_{16}$	nd	2.8 ± 2.8	58 ± 58	27 ± 19	80 ± 40
	p-Cymene	$C_{10}H_{16}$	nd	25 ± 17	9.5 ± 5.0	18 ± 14	6.2 ± 3.0
	Limonene	$C_{10}H_{16}$	134 ± 25	26 ± 12	31 ± 10	14 ± 3.5	22 ± 11
	β-Myrcene	$C_{10}H_{16}$	nd	nd	nd	8.6 ± 8.6	4.9 ± 4.7
	(*E*)-β-ocimene	$C_{10}H_{16}$	nd	99 ± 49	54 ± 51	37 ± 37	3.1 ± 2.8
	α-Pinene	$C_{10}H_{16}$	58 ± 21	69 ± 32	57 ± 19	5.0 ± 5.0	59 ± 30
	β-Pinene	$C_{10}H_{16}$	18 ± 7	12 ± 6.0	22 ± 6.5	3.2 ± 3.3	12 ± 6.5
	α-Phellandrene	$C_{10}H_{16}$	nd	nd	nd	0.16 ± 0.15	nd
	β-Phellandrene	$C_{10}H_{16}$	nd	4.3 ± 2.4	3.9 ± 2.4	2.4 ± 2.2	2.8 ± 1.4
	α-Terpinene	$C_{10}H_{16}$	11 ± 5	nd	0.15 ± 0.15	nd	nd
**Oxygenated monoterpenes**	(*E*)-Dihydrocarvone	$C_{10}H_{16}O$	12 ± 5	nd	nd	nd	nd
	Camphor	$C_{10}H_{16}O$	68 ± 11	nd	nd	nd	nd
	β-Cyclocytral	$C_{10}H_{16}O$	36 ± 16	nd	nd	nd	nd
	1,8-cineole	$C_{10}H_{18}O$	nd	0.49 ± 0.49	nd	nd	nd
	Linalool	$C_{10}H_{18}O$	32 ± 14	nd	nd	nd	nd
	α-Terpineol	$C_{10}H_{18}O$	18 ± 6	nd	nd	nd	nd
**Sesquiterpenes**	Cubebene	$C_{15}H_{24}$	26 ± 12	nd	nd	nd	nd
	(*E*)-β-Farnesene	$C_{15}H_{24}$	nd	40 ± 20	nd	5.1 ± 2.9	5.2 ± 2.7
	Longifolene	$C_{15}H_{24}$	nd	nd	nd	2.4 ± 2.4	nd
	Aromadendrene	$C_{15}H_{24}$	nd	nd	0.96 ± 0.96	nd	nd

nd: not detected
